# Microbial Communities Can Be Described by Metabolic Structure: A General Framework and Application to a Seasonally Variable, Depth-Stratified Microbial Community from the Coastal West Antarctic Peninsula

**DOI:** 10.1371/journal.pone.0135868

**Published:** 2015-08-18

**Authors:** Jeff S. Bowman, Hugh W. Ducklow

**Affiliations:** 1 Lamont-Doherty Earth Observatory, Columbia University, Palisades, New York, United States of America; 2 Blue Marble Space Institute of Science, Seattle, Washington, United States of America; Medical University Graz, AUSTRIA

## Abstract

Taxonomic marker gene studies, such as the 16S rRNA gene, have been used to successfully explore microbial diversity in a variety of marine, terrestrial, and host environments. For some of these environments long term sampling programs are beginning to build a historical record of microbial community structure. Although these 16S rRNA gene datasets do not intrinsically provide information on microbial metabolism or ecosystem function, this information can be developed by identifying metabolisms associated with related, phenotyped strains. Here we introduce the concept of metabolic inference; the systematic prediction of metabolism from phylogeny, and describe a complete pipeline for predicting the metabolic pathways likely to be found in a collection of 16S rRNA gene phylotypes. This framework includes a mechanism for assigning confidence to each metabolic inference that is based on a novel method for evaluating genomic plasticity. We applied this framework to 16S rRNA gene libraries from the West Antarctic Peninsula marine environment, including surface and deep summer samples and surface winter samples. Using statistical methods commonly applied to community ecology data we found that metabolic structure differed between summer surface and winter and deep samples, comparable to an analysis of community structure by 16S rRNA gene phylotypes. While taxonomic variance between samples was primarily driven by low abundance taxa, metabolic variance was attributable to both high and low abundance pathways. This suggests that clades with a high degree of functional redundancy can occupy distinct adjacent niches. Overall our findings demonstrate that inferred metabolism can be used in place of taxonomy to describe the structure of microbial communities. Coupling metabolic inference with targeted metagenomics and an improved collection of completed genomes could be a powerful way to analyze microbial communities in a high-throughput manner that provides direct access to metabolic and ecosystem function.

## Introduction

Biological communities are structured by a variety of physical, chemical, and ecological environmental factors. For the marine microbial community, these include the availability of dissolved organic carbon (DOC), the distribution of bioavailable nitrogen and phosphorous, light, and temperature, among numerous other biological, chemical, and physical factors. Although microbial community structure is often described in terms of taxonomy, with clear correlations between the taxonomic composition of various microbial communities and different environmental settings [1,2], these environmental conditions are more directly linked to metabolic structure. The cyanobacterial genus *Trichodesmium*, for example, is associated with the low concentration of bioavailable nitrogen in the tropical and subtropical oceans. It is the metabolic properties (e.g. diazotrophy) of the genus and not its taxonomy, however, that afford a direct link with environmental conditions. To understand the role of microbial communities in biogeochemical processes it is preferable to consider the metabolic structure of a community over its taxonomic structure.

The correlation between taxonomy and metabolic function [3,4] is the basis for the considerable body of work focused on the identification of community structure and composition through taxonomic marker gene analysis, namely the 16S rRNA gene. Although sometimes criticized as “stamp collecting” [5], these marker gene studies have enabled microbial ecologists to identify complex patterns of microbial diversity in a large number of geographic locations, and under widely varying environmental conditions. In contrast to the ease with which large 16S rRNA gene libraries can be generated however, it is not practical for a team of investigators to manually and exhaustively explore the metabolisms known to associate with all the observed operational taxonomic units (OTUs). More recently metagenomics studies, which profile not just a marker gene but all genes within a microbial community, have made it possible to explore total metabolic potential—though this type of analysis introduces another set of challenges, including cost, dataset size and throughput, and, in some cases the poor knowledge of gene function.

In a process analogous to the cellular organization of metazoans and plants, microbial communities partition metabolites and metabolic transformations within individuals, though this compartmentalization is imperfect. Although in some cases it is possible to reconstruct partial [6] or even full genomes [7] from metagenomics datasets, thus reproducing cellular partitions, this datatype does not lend itself to high confidence metabolic reconstruction; for example it is difficult to exclude possible chimeric metabolic pathways unless an investigator is very conservative and the assembly particularly robust. Furthermore, despite a precipitous decrease in the cost of sequencing since the first application of so-called next generation sequencing (NGS) to environmental samples in 2006 [8], it remains prohibitively expensive for most investigators to produce sufficiently deep, high-quality metagenomes on tens, let alone hundreds, of samples. As a result of these limitations new tools and a new conceptual framework are needed to bridge the gap between marker gene studies, which are economical but indirectly linked to community function, and potential metabolism, which is costly and labor intensive to analyze but is directly linked to function. Several recent studies have begun to make progress in this direction. Okuda et al. [9] undertook the construction of artificial metagenomes from collections of 16S rRNA sequences, finding a high degree of similarity between a reconstructed and actual metagenome. More recently Langille et al. [4] introduced an open-source tool, PICRUSt, to infer functional gene and pathway profiles from 16S rRNA gene data. In an extensive evaluation PICRUSt based predictions agreed well with the results of metagenomic analyses for diverse environments, including the human microbiome [4]. Together these studies suggest that metabolic inference from 16S rRNA gene libraries has a sound theoretical basis.

Despite the demonstrated success of this method there are limitations to how well microbial metabolism can be inferred from taxonomy. First, the connection between inferred metabolism and taxonomy is only as good as the collection of published sequenced genomes. At the time of analysis over 2,700 finished genomes were available on Genbank, along with more than 20,000 draft genomes. For strains that are very closely related to these genomes a reasonable inference of metabolism can be made, though incomplete, ambiguous, or incorrect annotations will lead to inaccuracies. Second, genomic plasticity is known to be high for many clades of the Bacteria and Archaea. Genetic composition, and thus metabolic potential, can vary widely between strains with a nearly identical 16S rRNA gene sequence [10]. Third, phenotypic plasticity means that even clonal strains encountering similar environmental conditions will drift in their specific metabolic response [11].

Here we present a novel framework for inferring complete metabolic pathways from 16S rRNA gene sequence collections, based on a phylogenetic placement approach [12] and the MetaCyc pathway ontology [13]. Our framework includes a method for quantifying genomic plasticity, which we apply to all finished genomes in the Genbank collection. We evaluated our metabolic inference methods against a marine metagenome, and applied both PICRUSt and our framework to 16S rRNA gene sequence libraries from the coastal West Antarctic Peninsula (WAP), inferring metabolic pathways in 18 samples separated by location, depth, and season. Both metabolic and taxonomic structure showed clear separation by sample type, suggesting that the abundance of metabolic pathways can be used in place of the abundance of OTUs to describe inter-sample relationships. This approach has the additional advantage of identifying the metabolisms most relevant to microbial community function in each environment. Although it is beyond the scope of the current work, our framework allows for a further analysis of community function by metabolic flux analysis [14]. This modeling of chemical conversions within and between cellular spaces has the potential to resolve connections between the composition of a microbial community and its ecosystem functions.

## Methods

### 16S rRNA gene reference tree construction

All 2,773 completed Bacterial and Archaeal genomes available on December 1, 2014 were downloaded from Genbank. The 16S rRNA genes were identified in each genome by blastn [15] search against the NCBI 16SMicrobial database. Although most of the analyzed genomes contained many 16S rRNA genes, sequence variation between these genes is thought to be minor [16]. Thus only the first identified 16S rRNA gene for each genome was used in downstream analysis. Extracted 16S rRNA gene sequences were aligned with Mothur v1.33.3 [17] against the Silva reference alignment available on the Mothur website (http://www.mothur.org/wiki/Alignment_database). Sequences shorter than 1,200 bases were discarded after alignment, and all sequences were then trimmed to the latest start and earliest end position. The 16S rRNA alignment was then used to build a phylogenetic tree with FastTree v2.1 [18] under the general time reversible (GTR) model. Using the software package Taxit [12] the 16S rRNA gene alignment and phylogenetic tree were then converted into a reference package for phylogenetic placement with pplacer [12].

### 16S rRNA gene analysis

We used the 16S rRNA gene sequences from Genbank SRA accession SRP016030 as reported in Luria et al. [19]. All 16S rRNA sequence analysis was carried out in Mothur v1.33.3 [17]. Reads were aligned to the Silva reference alignment as already described. All reads shorter than 100 bases, or that did not align to the region between positions 31,124 and 34,461 on the reference alignment, were discarded. The remaining reads were filtered to the latest start and earliest end position. Because the presence of chloroplasts would bias our analysis we removed all suspected chloroplasts after classifying the reads in Mothur against the Silva reference taxonomy available on the Mothur website at http://www.mothur.org/wiki/Silva_reference_files. Although no reads classified as “chloroplast” or “cyanobacteria,” we discovered that a large number of reads that did not classify below the level of Bacteria placed with the cyanobacterial genus *Cyanotheca*. Blastn search against the NCBI nt database confirmed that these were *Cryptomonad* chloroplast sequences. All reads that did not classify below the domain level were thus excluded from downstream analysis. Each sample was then randomly subsampled to 1,977 reads, the size of the smallest library. Subsampled reads from each sample were placed on the reference tree of 16S rRNA gene sequences from completed genomes using pplacer [12], keeping only a single placement. To describe placements on the reference tree we use the terms terminal node; meaning branch tip, internal node; meaning a point of bifurcation within the tree, and edge; meaning a path between two adjacent nodes. While the reference tree is composed of internal and terminal nodes, placements are made to edges. Edges are identified by the consensus taxonomy of the daughter nodes, or, if the edge leads to a terminal node, by the identity of the terminal node. Edge numbers given in the text and Table 1 refer to the edge numbers on the reference tree, provided as S1 File.

**Table 1:** Pathways with abundance greater than 60 appearing in only a single edge.

Pathway	Sample	Abundance	Edge	Taxa
glycine betaine degradation I	summer_sw_shallow_b.2	266	3324	*Candidatus Pelagibacter ubique* HTCC1062
glycine betaine degradation I	summer_sw_shallow_b.1	240	3324	*Candidatus Pelagibacter ubique* HTCC1062
glycine betaine degradation I	summer_se_shallow_b.2	237	3324	*Candidatus Pelagibacter ubique* HTCC1062
glycine betaine degradation I	summer_nw_shallow_b.2	167	3324	*Candidatus Pelagibacter ubique* HTCC1062
glycine betaine degradation I	summer_se_shallow_b.1	142	3324	*Candidatus Pelagibacter ubique* HTCC1062
NAD biosynthesis III	summer_nw_deep_b.2	111	268	*Francisella* spp.
glycine betaine degradation I	summer_ne_shallow_b.1	105	3324	*Candidatus Pelagibacter ubique* HTCC1062
kojibiose degradation	summer_nw_shallow_b.2	87	660	*Muricauda ruestringensis* DSM 13258
phosphonoacetate degradation	summer_sw_shallow_b.2	83	3456	*Glaciecola nitratireducens* FR1064
tryptophan degradation VI	summer_nw_shallow_b.1	78	201	*Teredinibacter turnerae* T7901
acrylonitrile degradation II	summer_nw_shallow_b.1	78	201	*Teredinibacter turnerae* T7901
nitrate reduction IV (dissimilatory)	summer_ne_shallow_b.2	73	1242	*Syntrophomonas wolfei* Goettingen
urate degradation to allantoin I	summer_sw_shallow_b.1	67	3095	*Octadecabacter* spp.
pyruvate fermentation to ethanol III	summer_nw_deep_b.1	67	201	*Teredinibacter turnerae* T7901
L-rhamnose degradation II	summer_sw_shallow_b.1	67	3095	*Octadecabacter* spp.
arginine degradation IV	summer_nw_deep_b.1	67	201	*Teredinibacter turnerae* T7901
acrylonitrile degradation II	summer_nw_deep_b.1	67	201	*Teredinibacter turnerae* T7901
phenylalanine degradation V	summer_sw_deep_b.1	66	3480	*Colwellia psychrerythraea* 34H
acrylonitrile degradation II	summer_sw_shallow_b.2	66	201	*Teredinibacter turnerae* T7901
phenylalanine degradation V	summer_sw_deep_b.2	65	3480	*Colwellia psychrerythraea* 34H
isopenicillin N biosynthesis	summer_ne_shallow_b.1	64	268	*Francisella* spp.

### Genome database construction

We considered two scenarios for inferring genomic composition from read placement on our reference tree. In the first scenario a query read placed to an edge leading to a terminal node on our reference tree; in this case the inferred genome was simply the genome of the reference sequence defining the terminal node. In the second scenario a query read placed to an edge connecting internal nodes on our reference tree. In this case we inferred the genome as all genes that were shared between all members of the clade rooted at the internal node. To identify these genes we traversed the reference tree using the Phylo package in Biopython [20]. For each internal node we generated a blast database of the coding sequences (CDS) for one genome (including all genetic elements), and used the discontiguous-megablast task in blastn to search the CDS of all other genomes against it, with an e-value cutoff of 1. The genome used to build the reference database was then trimmed to include only those CDS present in all clade members. This trimmed genome is referred to as the core genome.

### Accounting for uncertainty

Although we have no way of accounting for phenotypic plasticity in our method, we account for genomic variability in two ways. First, we quantified the size of each core genome compared to all members of each clade. For very genetically stable clades even deeply rooted nodes will have a core genome that approaches the mean genome size for all clade members. Conversely, in clades with a high degree of genomic plasticity even shallow nodes have a small core genome relative to the mean genome size. Second, because genomic stability cannot be estimated from comparisons of genome size for terminal nodes, we evaluated the relative genetic plasticity of all reference genomes by comparison of distance calculated on proteome compositional vectors and 16S rRNA genes. A larger difference between these two distances, for a given strain in comparison with all other strains, suggests a more divergent genome relative to the 16S rRNA gene. Such divergence may have been brought about by a high degree of gene acquisition, loss, or duplication.

To generate the compositional vectors we followed the method of Qi et al. [21], which relies on 5 amino acid length kmers. Due to memory limitations we found distance matrix calculation on vectors of 20^5^ kmers impractical, and applied a dimension-reduction step to identify those kmers most responsible for variability between proteomes. For dimension reduction 200 genomes were selected at random. Principal component analysis (PCA) was carried out on a transformed matrix of compositional vectors for these strains, such that 200 principal components (PCs) were calculated. Examination of a scree plot indicated that it was necessary to consider the first 163 PCs to describe 90% of the variance. We then considered the sum magnitude of each kmer for these 163 PCs, normalizing the magnitude to the proportion of variance for each PC. Examination of a second scree plot, of total magnitude by kmer, showed a clear inflection point at approximately 100,000 kmers. Bray-Curtis distance was then calculated on both the full-length compositional vectors and partial compositional vectors for the 200 strains. Pearson’s correlation coefficient was used to describe agreement between the two calculations (S1 Fig).

For all strains a matrix of 16S rRNA gene distances was generated using Mothur [17] on our existing alignment. Both the 16S rRNA and partial compositional vector distance matrices were transformed to have a mean of 0 and a variance of 1, and then scaled to be in the range 0 to 1. We found that following normalization 16S rRNA distance (d_16_) could be described as a function of partial compositional vector distance (d_cv_) according to an exponential function (R^2^ = 0.35, S2 Fig):

$$d_{16}=5*10^{-6}e^{12.523d_{cv}}$$ (1)

Using this relationship we calculated the residual (predicted—observed) between the predicted and observed 16S rRNA gene distances. Positive residuals indicate 16S rRNA genes for which the phylogenetic distance is less than would be expected, given the difference in proteome composition. We take this as an indicator of high genomic plasticity in one of the two stains in the pairwise comparison. We then took the mean across rows of the resulting matrix of residuals (S3 Fig), and transformed the distribution of means to the range 0 and 1. These transformed mean values (ϕ) provide an index of genetic stability for each of the genomes in our analysis.

We combined our measures of uncertainty to produce a metric that describes the relative quality (q) of our metabolic predictions, where S_core_ = the size of the core genome and S_clade_ = the mean genome size of the clade:

$$q=\;\frac{S_{core}}{S_{clade}}*\left(1-\;\phi \right)$$ (2)

Note that for terminal nodes the first term is assumed to be 1. For each sample, the mean score of the predicted genomes is our quality score for that sample.

### Genome selection and pathway creation

For each sample the phylogenetic placements were used to select core genomes. Metabolic pathways were predicted for each core genome with a placement using the Pathologic software included with Pathway Tools v. 18.5 [22]. The pathways-report.txt file created by Pathologic and read placement abundance data were used to generate an abundance matrix of pathways for each sample. Further comparative analyses between samples were carried out using R [23]. All scripts necessary to reproduce the database construction, phylogenetic analysis, and core genome selection can be obtained from http://github.com/bowmanjeffs/genome_finder.

### Metagenomic analysis

A marine metagenome collected near the South Orkney Islands at 62.14°S and 62.04°W during the *Tara* Oceans expedition [24] from 10 m depth on 6 January 2011 was downloaded from the Genbank SRA (accession ERR599176). The first and last 10 positions were trimmed from each read, and reads with a mean quality score below 20, with 5 or more bases below 20, shorter than 100 bases in length, or with more than 5 homopolymers were discarded. 22,741,715 reads passed quality control. QC’d reads associated with 16S rRNA gene sequences were identified by blastn megablast search against the 16SMicrobial database with an E-value cutoff of 10^−10^. This search identified 31,267 putative 16S rRNA reads that were used to predict pathways with PICRUSt and with our method.

To predict pathways directly from metabolic genes represented in the metagenome we used DIAMOND blastx [25] to search all reads against a protein database constructed from all translated coding sequences in the completed Genbank genomes. New Genbank format annotation files were constructed for each genome containing only those features identified as hits in the DIAMOND search. Pathways were predicted for each Genbank file using Pathologic [22] as described previously. This method has the advantage of mimicking the cellular partitioning of genes into pathways, but, because coverage of the true metagenome by the sequenced metagenome is variable and incomplete [4], it likely underestimates pathway richness. To provide an alternate estimate of pathway richness we also constructed pathways without cellular partitions by generating an artificial genome with a CDS representing each unique BRENDA EC number [26] and gene product identified in the metagenome.

### PICRUSt

PICRUSt metabolic predictions were carried out on closed OTUs at the 97% similarity level. OTUs were assigned with QIIME [27] against Greengenes v13.5 [28]. Metabolic predictions on copy-number normalized OTUs were made through the Galaxy [29] server located at http://huttenhower.sph.harvard.edu/galaxy/.

## Results and Discussion

### Genetic plasticity

**Fig 1:**
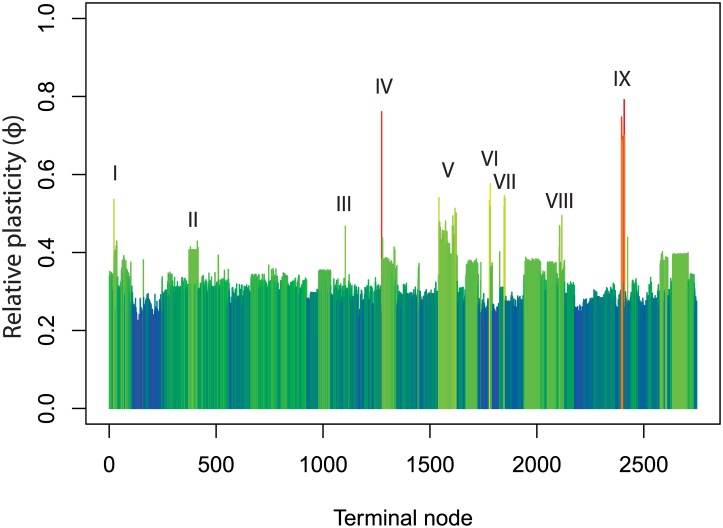
Relative genomic plasticity of the genomes used for metabolic inference. Colors correspond to the level of plasticity, which is also given by the y-axis. X-axis is labeled according to terminal node order for the representative 16S rRNA gene from each genome on a phylogenetic tree rooted at the node ancestral to the Archaea and *Planctomyces* (S1 File). Note that terminal node order on this rooted tree does not correspond to edge numbers on the unrooted tree used for phylogenetic placement (S2 File). Arrows indicate strains or clades with exceptional plasticity, the majority of which are known symbionts. I) *Nanoarcheum equitans* II) the *Mycobacteria* III) a butyrate producing bacterium within the *Clostridium* IV) *Candidatus Hodgkinia circadicola* V) the *Mycoplasma* VI) *Sulcia muelleri* VII) *Portiera aleyrodidanum* VIII) *Buchnera aphidicola*, IX) the *Oxalobacteraceae*.

The comparison of 16S rRNA gene and compositional vector distance revealed distinct patterns of genomic plasticity across the analyzed genomes (Fig 1). The most unstable genomes in our analysis belonged to a clade of insect symbionts that include *Candidatus Tremblaya princeps* PCVAL, *Oxalobacteraceae* symbiotic bacterium CARI, *Candidatus Nasuia deltocephalinicola* NAS ALF, and *Candidatus Carsonella ruddii*. This high degree of instability is consistent with expectations for obligate symbionts, which often have highly streamlined genomes [30] and high rates of horizontal gene transfer [31]. Other regions of high genomic plasticity were also dominated by symbionts, including *Candidatus Hodgkinia cicadicola* DSEM, *Candidatus Sulcia muelleri*, *Candidatus Portiera aleyrodidarum*, *Buchnera aphidicola*, and *Nanoarchaeum equitans*. The *Mycobacteria* and *Mycoplasma* also had exceptional levels of genomic plasticity.

### Comparison with metagenome

**Fig 2:**
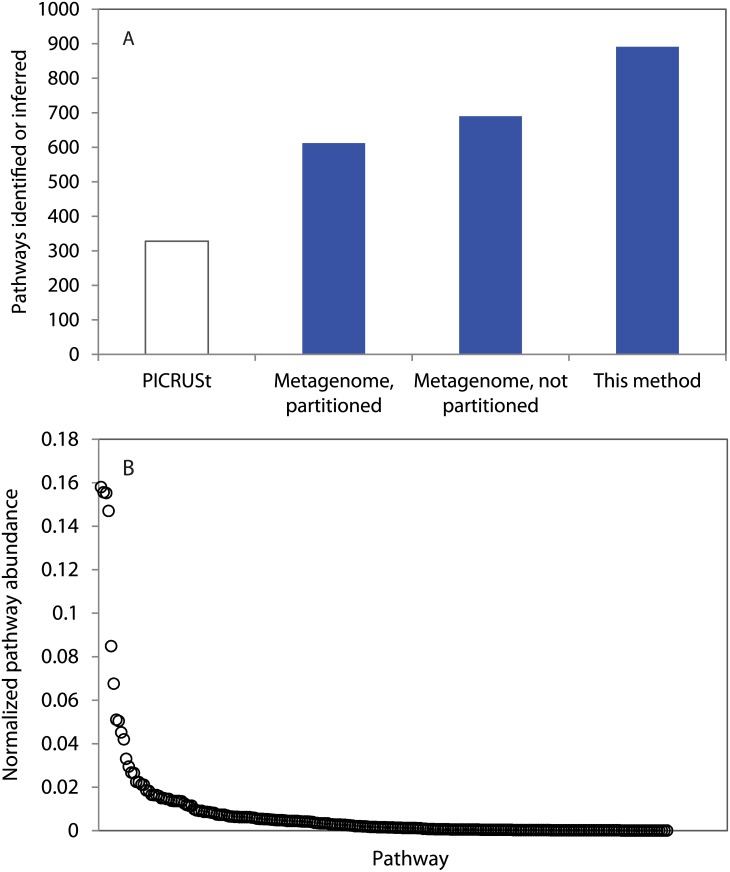
Comparison of metabolic inference and metagenomic analysis. A) Metabolic pathways identified (metagenomes) or inferred (PICRUSt, this method) with each method. PICRUSt pathways (indicated by white bar) are based on the KEGG ontology, thus the number of pathways inferred is not directly comparable to the other methods which are based on the MetaCyc ontology. B) The abundance of pathways inferred for but not identified in the metagenome.

Our metabolic inference method predicted 891 metabolic pathways for the South Orkney Islands metagenome. A lesser number of pathways were identified directly from functional genes represented in the metagenome; 612 and 690 for the partitioned and non-partitioned methods respectively. Most of the 251 pathways predicted by metabolic inference, but not identified in the metagenome, were associated with low abundance taxa (Fig 2) suggesting that their absence may be the result of incomplete coverage. Several of these pathways however, had an abundance greater than 4,500 (14.4% of 16S rRNA read abundance), including the pentose phosphate pathway (oxidative branch) II, indole-3-acetate biosynthesis V (bacteria and fungi), lysine degradation I, and cysteine biosynthesis/homocysteine degradation. The greater abundance of these pathways in the metabolic inference does not necessarily preclude incomplete coverage of the metagenome as a reason for their absence in that dataset, alternatively, they may represent pathways inferred for but not present within the sampled community.

Metabolic inference with PICRUSt yielded 328 metabolic pathways for the metagenome, however, it is important to recognize that our PICRUSt analysis relied on the Kyoto Encyclopedia of Genes and Genomes (KEGG) ontology [32] which is not directly comparable to the MetaCyc ontology [13], nor are we aware of any method for converting between ontologies. Thus the lower number of predicted pathways for PICRUSt is not a reflection of lower sensitivity, but it may reflect a lesser degree of specificity in the final result.

### Application to marine samples from the WAP

**Fig 3:**
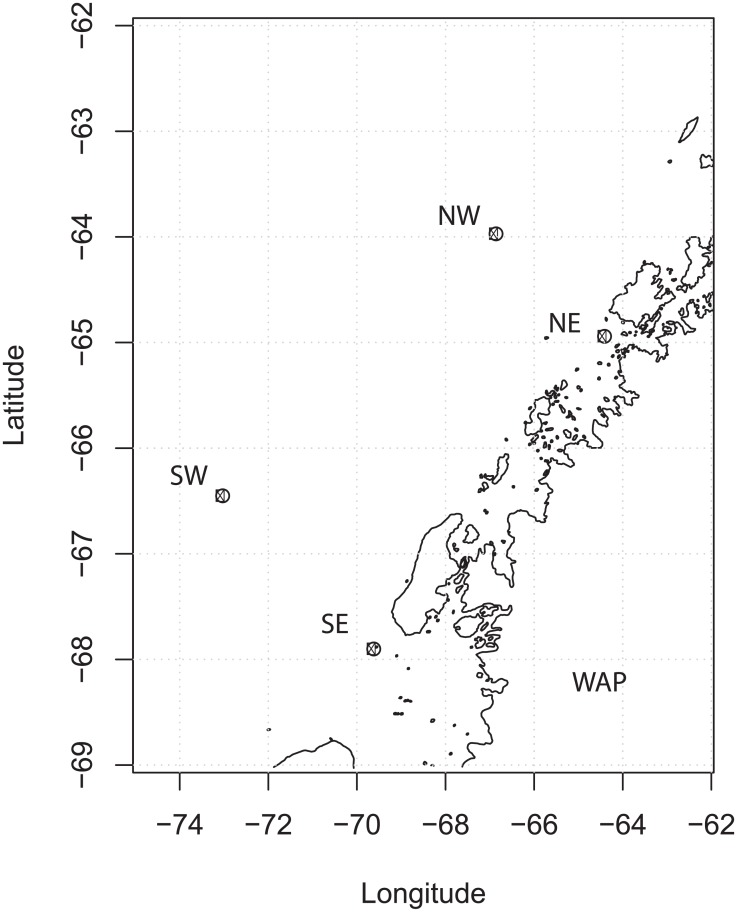
Sample locations in the West Antarctic Peninsula (WAP). Summertime surface (10 m) and deep (100 m) samples are analyzed from two inshore and two offshore samples, organized along a North to South gradient. Winter surface water samples were analyzed from the northern, inshore station (NE).

To test our metabolic inference method on 16S rRNA gene amplicon datasets we selected a sample set obtained from the Palmer Long Term Ecological Research site (PAL-LTER) off the WAP [19] (Fig 3). Using an OTU based approach on these samples, Luria et al. [19] had previously found that samples from like environments grouped together by hierarchical clustering and nonmetric multidimensional scaling (NMDS), although there was significant variation between samples along all gradients (north vs. south, inshore vs. offshore, surface vs. deep). Inshore surface samples, which also host large populations of eukaryotic *Cryptomonads* [19], showed the largest difference from other sample types. Winter surface samples had some similarity to deep summer samples, supporting the hypothesis that remnant “winter water” drives community structure at depth [19].

**Fig 4:**
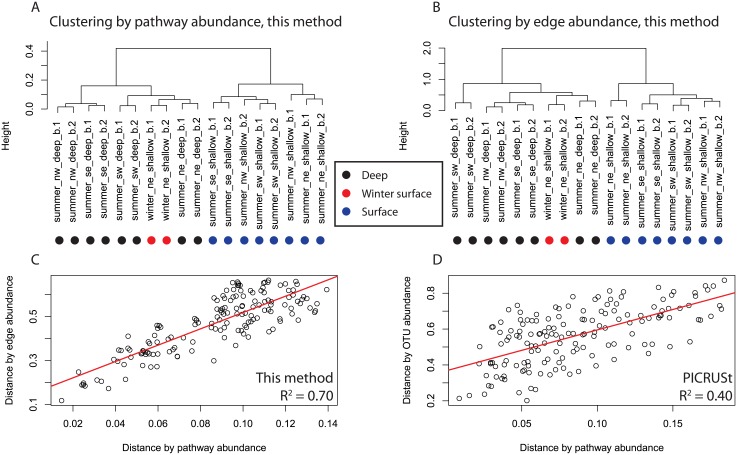
Hierarchical clustering of samples by pathway and edge abundance. Clustering used the Ward algorithm on Bray-Curtis distance. A) Hierarchical clustering by pathway after normalization to the maximum abundance of each pathway. B) Hierarchical clustering by edge abundance after normalization to the total abundance of each sample. C) Linear model (red line) for distance by edge abundance as a function of distance by pathway abundance, R^2^ = 0.65, df = 75, p ≈ 0. D) Linear model (red line) for distance by OTU abundance as a function of distance by pathway abundance, as predicted using PICRUSt (see text) [4].

**Fig 5:**
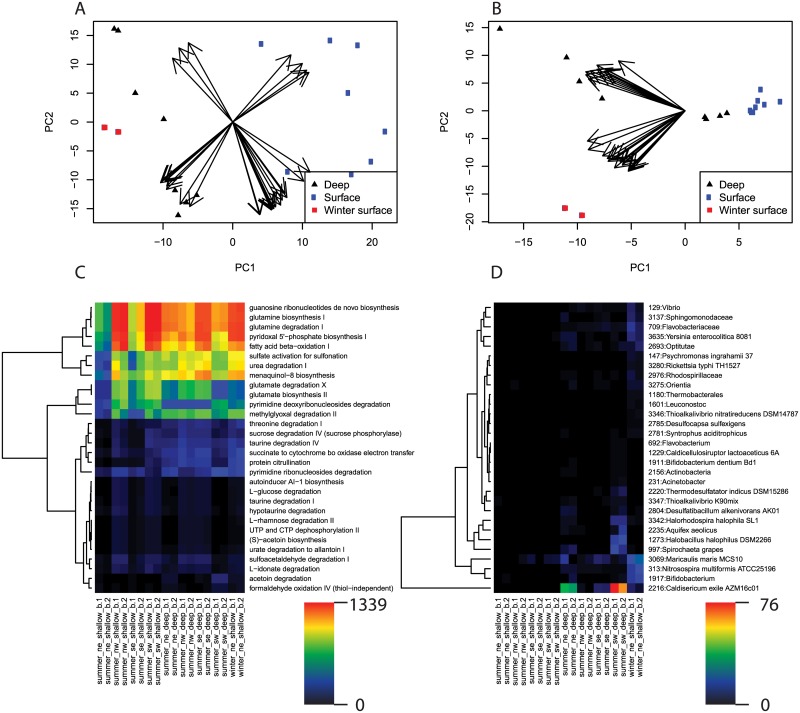
Metabolic pathways and edges accounting for the most variance between samples. A) PCA of normalized metabolic pathway abundance (see text). Arrows are vectors for the top twenty pathways ordered by their magnitude in PC1 and PC2. B) Heatmap of abundance for the top twenty pathways ordered by their magnitude in PC1 and PC2. Hierarchical clustering on the y-axis uses the defaults of the heatmap command in R; Euclidean distance and the complete linkage clustering method. C) PCA of normalized edge abundance (see text). Arrows are vectors of the top twenty edges ordered as for A. D) Heatmap of abundance for the top twenty edges ordered as for B. Hierarchical clustering on the y-axis is as for B. Values on the color bar are raw (not normalized) values.

Our phylogenetic placement approach to evaluating community structure broadly supports the findings of Luria et al. [19], however, we observed a clearer separation between winter and deep samples and summer surface samples (Fig 4), suggesting that a phylogenetic placement approach can improve the sensitivity of β-diversity analysis. To identify what edges were most responsible for variation between samples we applied PCA to our edge abundance data (Fig 5). Variance was primarily driven by low-abundance taxa; for the top 30 edges ranked by magnitude in PC1 and PC2 the most abundant edge in any one sample was edge 2216 (*Caldisericum exile* AZM16c01), at 76 occurrences in summer_sw_deep_b.1 (5.7% of the total population). Much of the variance between samples was driven by rare edges occurring more often in winter and deep samples than summer surface samples, including 2216 (*Caldisericum exile* AZM16c01), 1917 (*Bifidobacterium*), 313 (*Nitrosospira multiformis* ATCC25196), and 3069 (*Maricaulis maris* MCS10). Consistent with this we found the winter and deep samples to have greater predicted edge richness by Chao1 [33] as determined with a Mann-Whitney test (Chao1: mean summer surface = 118.9, mean winter and deep = 197.3, w = 13, p = 0.016). This increase in richness is consistent with other studies that observed greater richness in high latitude winter samples [19,34,35]. Overall the most abundant edges across all samples were 3324 (*Candidatus Pelagibacter ubique* HTCC1062), which comprised up to 22.3% of the total community (mean = 15.8%, sd = 4.0%), and 268 (an ancestral node to the Gammaproteobacterial clade *Franscella*), which comprised up to 15.6% of the total community (mean = 11.6%, sd = 3.0%).

Clustering by pathway abundance agreed strongly with clustering by edge abundance, with the pairwise distance between all sample strongly correlated (R^2^ = 0.70, p ≈ 0) and only minor differences in cluster membership (Fig 4A–4C). The correlation between samples by PICRUSt metabolic inference was weaker than with our method, but was still highly significant (R^2^ = 0.40, p ≈ 0) (Fig 4D). Unlike in the description of community structure by edge abundance however, the pathways accounting for the most variance between samples were not necessarily low in abundance, with high variance pathways falling into high, middle, and low abundance groups (Fig 5).

**Fig 6:**
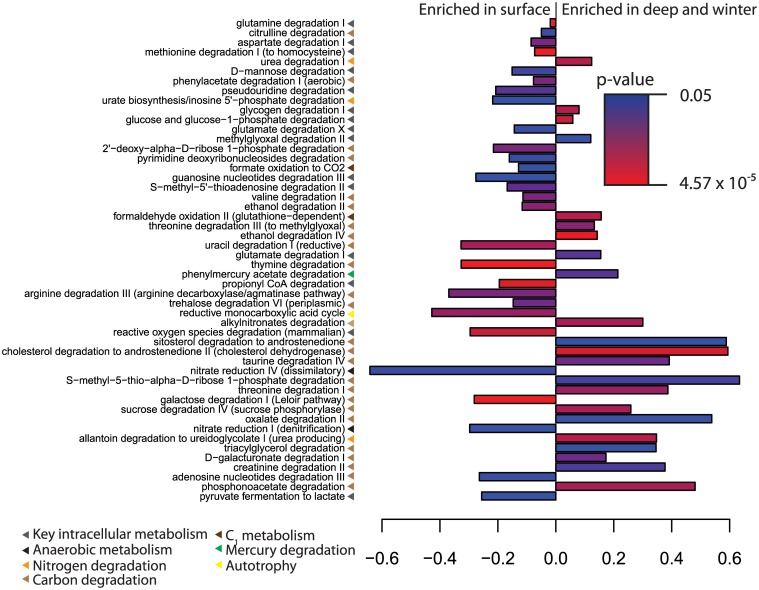
Metabolic pathways differentially present between summer surface samples and winter and deep samples. Color gives the p-value for a Mann-Whitney test between sample groups. X-axis gives the anomaly, calculated as the difference in sample group means divided by the sum of the sample group means.

To further explore the implications of metabolic pathway distribution on microbial ecosystem function we considered pathways involved in the degradation of carbon substrates and in energy acquisition, evaluating the normalized abundance of these pathways as a standard anomaly (the difference between summer surface and winter and deep samples divided by the normalized abundance in both groups) (Fig 6). Some pathways involved in the degradation of plausible DOC components were differentially present between groups, including pathways for the degradation of phenylacetate, ethanol, oxalate, amino acids, and nucleosides and their derivatives. A differential distribution of degradative pathways is consistent with the structuring of microbial communities around the composition of the DOC pool, a concept that has been explored in numerous studies [36–38]. Interestingly, two nitrate reduction pathways were present at a greater normalized abundance in the summer surface samples. Anaerobic environments are known to form in particles and aggregates in the otherwise oxic photic zone [39]. In the WAP these processes could be linked to anaerobic microenvironments that form during periods of high respiration and suggest an additional sink for nitrate.

Metabolic pathway abundance and Choa1 richness were not significantly different between summer surface and winter and deep samples by the Mann-Whitney test at the 95% confidence level (abundance: mean summer surface = 184,852, mean winter and deep = 208,951, w = 40, p = 1; Chao1: mean summer surface = 640.29, mean winter and deep = 674.57, w = 20, p = 0.08). Sample quality scores were significantly higher for the summer surface than winter and deep samples (mean summer surface = 0.61, mean winter and deep = 0.56, w = 67, p = 0.02), suggesting that the observed disparity in taxonomic and metabolic diversity between seasons and depths could be influenced by lower quality metabolic inferences for the winter and deep samples. This finding makes sense in the context of historical sampling; there is a sampling bias toward the surface ocean and, for high latitude waters, the summer season. Genomes from Bacteria and Archaea that are specialists below the photic zone and in dark winter surface waters are underrepresented in culture collections and in the database of completed genomes.

### Functional redundancy

**Fig 7:**
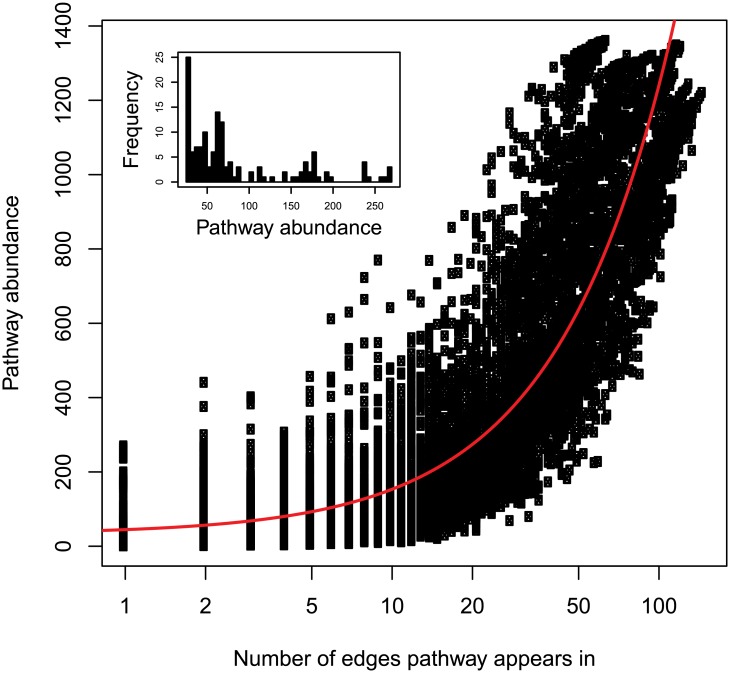
Pathway abundance as a function of the number of edges pathway appears in for a given sample. Inset is a histogram for abundance at the edge richness of 1 (i.e. the abundance of pathways with the lowest redundancy). Abundance and edge richness are linearly correlated (red line; R^2^ = 0.78, df = 11,623, p ≈ 0).

The region of the WAP is undergoing rapid climatic and ecological change [40]. Although it is not clear what impact this change will have on Bacterial and Archaeal communities, or even how persistent these communities are from year to year in the absence of rapid environmental change, changes to phytoplankton community structure suggest that microbial communities are prone to shift with the changing environment [41]. Of particular interest in our analysis are pathways that are present in very few strains, suggesting low functional redundancy. These pathways—and their corresponding ecosystem functions—could be lost, at least temporarily, if environmental conditions change rapidly. Examples of such rapid environmental change include deep mixing events, rapid glacial outflow, and the rapid retreat or advance of sea ice. A recent example of the rapid loss of a microbial ecosystem function was described by Steinle et al. [42] over the Arctic continental shelf. There, a rapid change in the structure of the water column temporarily eliminated a methanotrophic community associated with seafloor methane seeps. After 11 days, and despite the fact that the methanotrophic niche remained open, that particular function had not been fully restored to the ecosystem. To identify pathways in these samples that might have high ecological importance but low functional redundancy we considered abundant pathways that were present in very few genomes (edges) in any one sample (Fig 7). Although pathway abundance is an imperfect proxy of environmental significance as reaction rates, expression rates, and the concentration of substrates and products required to achieve significance are all independent of abundance, abundance is an indicator of *potential* significance. Low redundancy, high abundance pathways suggest metabolisms that could connect to major ecosystem shifts.

Across all samples 140 pathways were represented by only one edge. The fewer edges a pathway was predicted in the higher the likelihood that the pathway was predicted incorrectly, thus some of these nonredundant pathways are erroneous and not expected in the domain Bacteria. One example is the glycerol-3-phosphate shuttle, a mitochondrial energy carrier that is not expected outside the Eurkaryota, but that appears as a nonredundant pathway in 10 of 18 samples due to a prediction for edge 3324 (*Candidatus Pelagibacter ubique* HTCC1062). Although the logic for the prediction is sound; the annotation for the *Candidatus Pelagibacter ubique* HTCC1062 genome includes proteins with the BRENDA numbers EC 1.1.5.3 and EC 1.1.1.8, the requisite enzymes for this pathway, within the Bacteria these enzymes function in different but biochemically related processes. This overprediction highlights one of the current limitations of the method. Methodological improvements, including improved pathway specificity, will be priorities in future work. Aside from these probable erroneous classifications, we identified several nonredundant pathways of potential ecological significance (Table 1). These included glycine betaine degradation (edge 3324, *Candidatus Pelagibacter* ubique HTCC1062), isopenicillin N biosynthesis (edge 268, *Francisella* spp.), acrylonitrile degradation (edge 201, *Teredinibacter turnerae* T7901), and nitrate reduction IV (edge 1242, *Syntrophomonas wolfei* Goettingen).

### Conclusions

We’ve described a framework for inferring microbial metabolic structure, as defined by the abundance of metabolic pathways, from 16S rRNA gene data using a phylogenetic placement approach [12] and the MetaCyc ontology [13]. This metabolic inference framework is complementary to metagenomic analysis, and should be paired with genome sequencing and metagenomics to reasonably constrain the metabolisms present in any environment. Although metagenomics can provide a less biased profile of potential metabolism, insufficient metagenomic sequencing depth or highly variable coverage will make for an incomplete metabolic profile [4]. In addition we argue that it is neither necessary nor desirable to produce detailed, high quality metagenomes in a high-throughput fashion (i.e. on hundreds or thousands of samples required for a single ecological study). Even with the limited number of completed genomes currently available, however, it is possible to infer metabolism to a level that closely reproduces patterns observed by 16S rRNA gene comparison. Furthermore, by converting 16S rRNA gene community data directly to metabolic structure data, differences between samples can be evaluated in the context of changes to the ecosystem function of the microbial community. Although our use of complete pathways to describe metabolism is necessarily conservative, as a metabolism cannot be predicted unless a complete pathway has been described, presentation of the data in this fashion makes it most compatible with other ‘omics analyses. Gene expression and metabolite concentrations, for example, can be readily mapped to the PGDBs by available tools [22].

As with other metabolic inference techniques, the framework introduced here is only as good as the collection of completed genomes available in the public repositories and our knowledge of gene function. Although draft genome assemblies from metagenomes and single cell sequencing efforts have allowed investigators to access much of the metabolic potential of (as yet) unculturable marine microbes, few investigators take the time to complete the difficult task of closing the draft genomes produced by these analyses. Although our framework could be easily modified to include draft genomes, which would result in improved taxonomic resolution, the resulting inference would be less informative than if the genomes were complete. This is due to the low quality scores such an analysis would produce, since genomic plasticity would be artificially elevated and core genome size would be artificially small. Second, geographical variations in the proportion of microbial dark matter (the functionally uncharacterized portion of the microbial assemblage) [43] and in the degree of genomic plasticity require validation with thorough, high quality metagenomes. Differences between a metabolic inference and metagenomics analysis would highlight clades that are not well represented by sequenced genomes, or that are exceptionally plastic (and thus require a high degree of taxonomic resolution). These clades could then be targeted for isolation, genome sequencing, and phenotyping, or for genome assembly from a deep, targeted metagenome.

## Supporting Information

S1 Fig Correlation between distance from reduced compositional vectors and full compositional vectors for 200 randomly selected genomes. (PDF)

S2 Fig 16S rRNA gene distance as a function of reduced compositional vector distance. (PDF)

S3 Fig Square matrix of normalized residuals. Each row (or column) is a published complete genome. Row means (Fig 2) are a measure of genomic plasticity. White color is centered on the mean for the normalized residuals of 0.32. Blue values are pairwise comparisons where the predicted 16S rRNA gene distance was less than the observed, red values are pairwise comparisons where the predicted 16S rRNA gene distance was greater than the observed.
(PDF)

S1 File Unrooted tree used for phylogenetic placement. The tree shown is for sample summer_sw_shallow_b.1, edges are fattened to show the relative number of placements to each edge. The tree is in the PhyloXML format.
(ZIP)

S2 File Rooted reference tree without numbered edges. Terminal node order on this tree corresponds to the order given in Fig 1. Tree is in the Newick format and can be opened with a standard tree viewer.
(ZIP)

S3 File Tab-delimited file of pathways predicted for each sample. (TXT)
