# Supplementary figures and images for: Microbial Communities Can Be Described by Metabolic Structure: A General Framework and Application to a Seasonally Variable, Depth-Stratified Microbial Community from the Coastal West Antarctic Peninsula
Source: PLoS One. 2015 Aug 18;10(8):e0135868. doi: 10.1371/journal.pone.0135868 (PMC4540456; doi:10.1371/journal.pone.0135868)

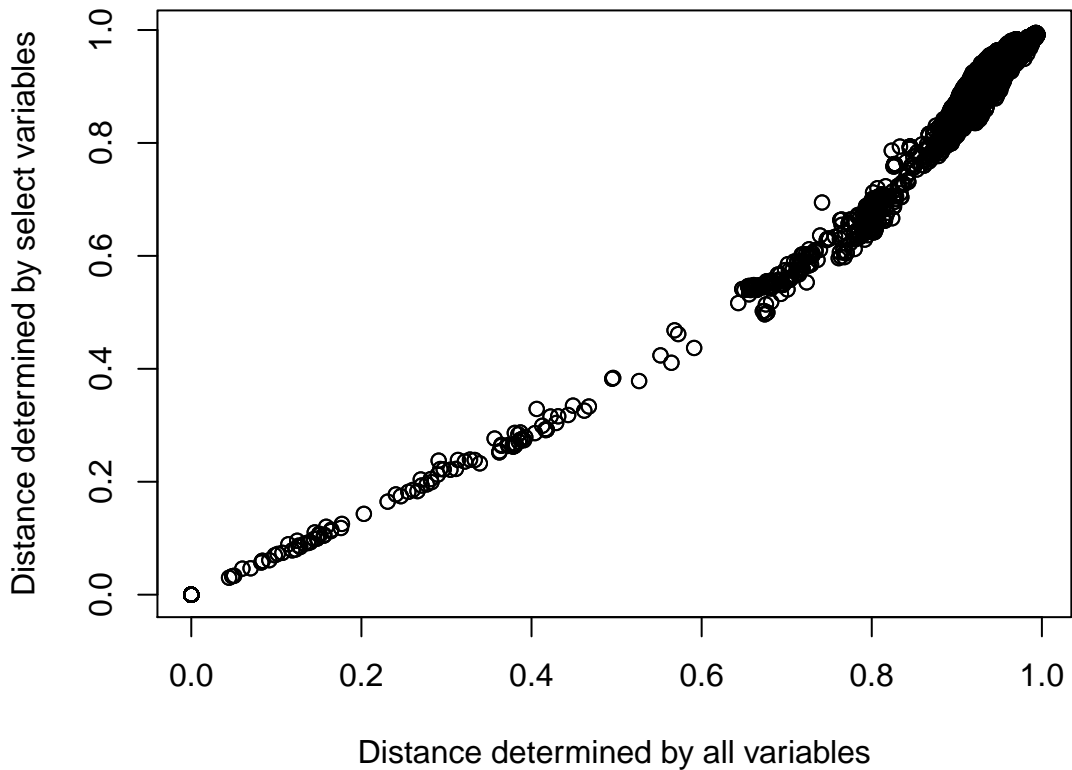

Supplement: S1 Fig — (PDF) [file pone.0135868.s001.pdf]

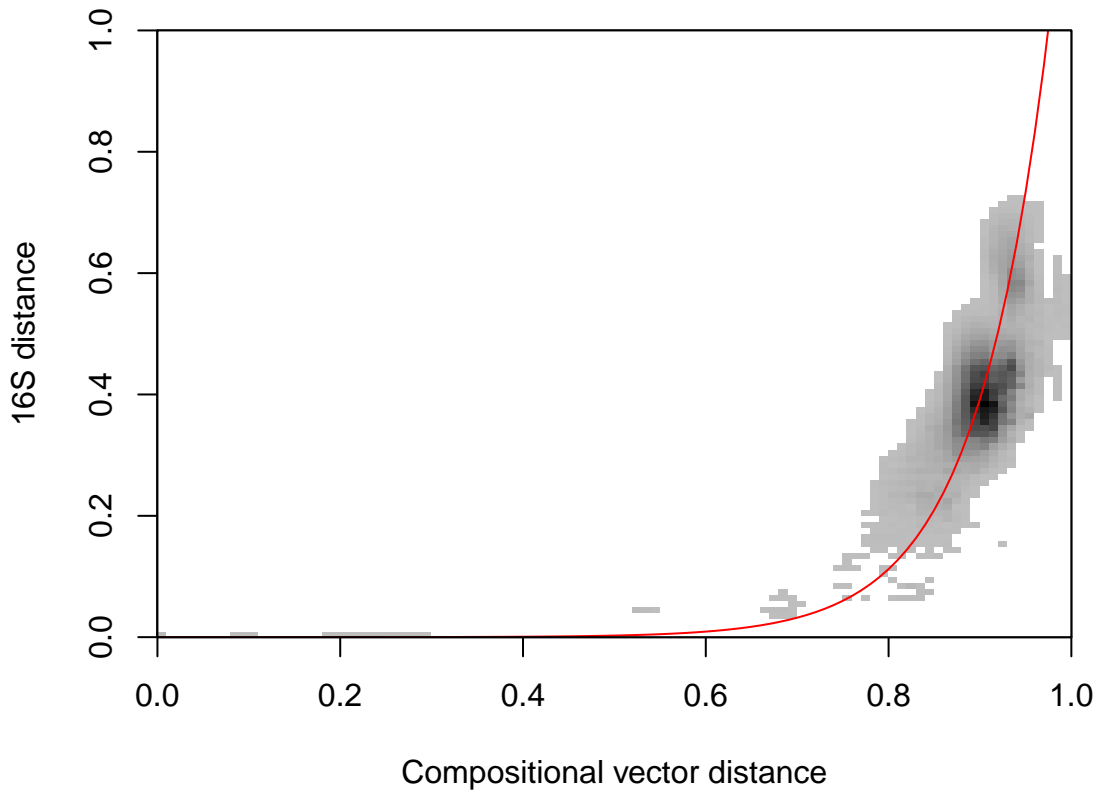

Supplement: S2 Fig — (PDF) [file pone.0135868.s002.pdf]

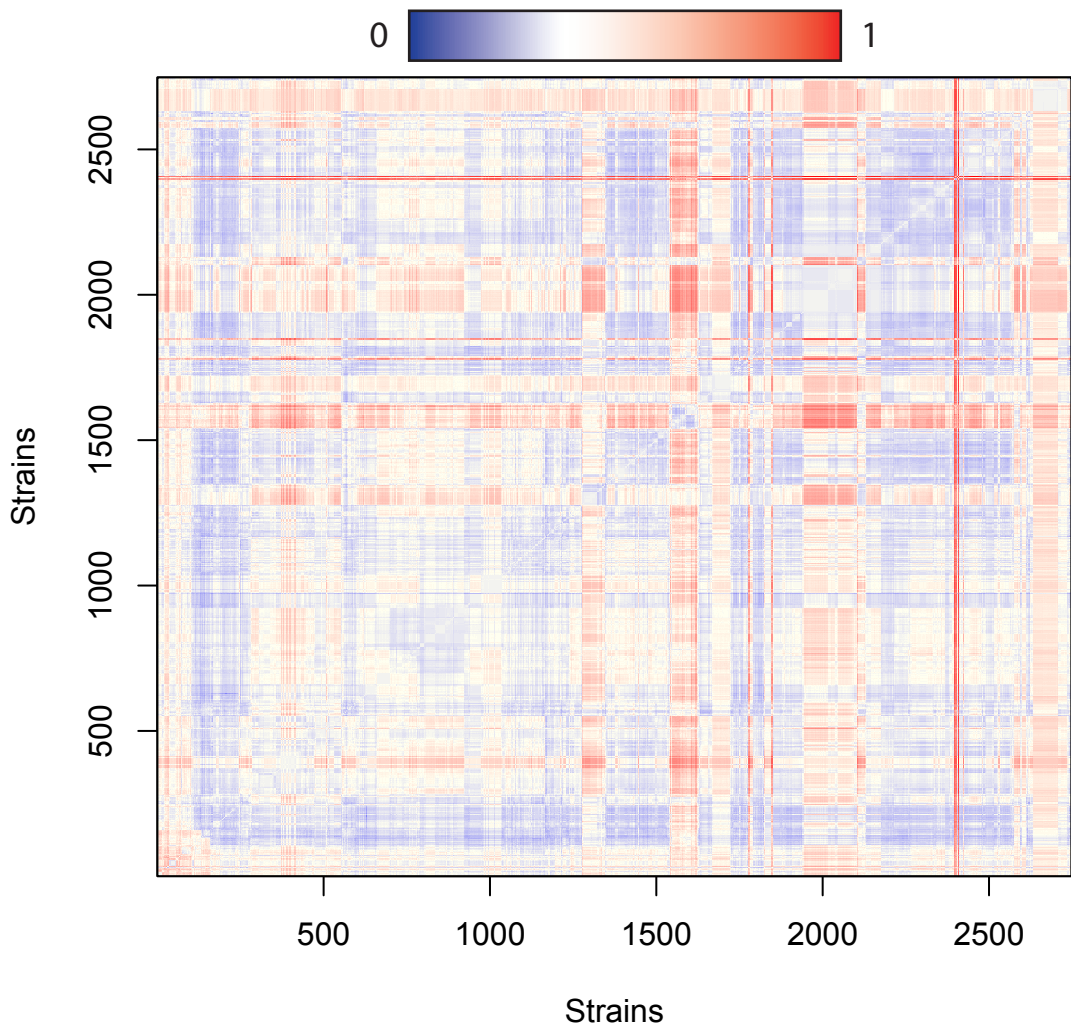

Supplement: S3 Fig — Each row (or column) is a published complete genome. Row means (Fig 2) are a measure of genomic plasticity. White color is centered on the mean for the normalized residuals of 0.32. Blue values are pairwise comparisons where the predicted 16S rRNA gene distance was less than the observed, red values are pairwise comparisons where the predicted 16S rRNA gene distance was greater than the observed. (PDF) [file pone.0135868.s003.pdf]
